# Hydrogen Sulfide-Releasing Compounds Attenuate Lipopolysaccharide-Induced Inflammation in Porcine Anterior Uveal Explants, Ex Vivo

**DOI:** 10.21203/rs.3.rs-9698771/v1

**Published:** 2026-05-25

**Authors:** Anthonia Okolie, Fatima Muili, Rahel Isak, Sunny E. Ohia, Catherine A. Opere, Ya Fatou Njie Mbye

**Affiliations:** Texas Southern University; Texas Southern University; Texas Southern University; Texas Southern University; Creighton University; Texas Southern University

**Keywords:** hydrogen sulfide, inflammation, uveitis, anterior uvea, cytokine

## Abstract

In the present study, we investigated the pharmacological actions of both fast- and slow-releasing H_2_S compounds, as well as polysulfides, on acute inflammation induced by the bacterial lipopolysaccharide (LPS) in an *ex vivo* porcine model of anterior uveitis. Isolated porcine iris-ciliary bodies (ICB) were maintained in oxygenated Krebs-Henseleit solution and then incubated in separate wells of RPMI 1640 supplemented media with an antibiotic. ICBs were exposed to sodium hydrosulfide (NaHS), GYY4137, S-allyl-cysteine (SAC), and polysulfides, diallyl disulfide (DADS), and diallyl tetrasulfide (DATTS) four hours before the end of incubation. We observed that LPS (5–200 ng/ml) produced increases in the release of pro-inflammatory mediators (TNF-α, IL-6, and PGE_2_) and a corresponding decrease in the anti-inflammatory marker, IL-10, in the ICB explants. Both fast- (NaHS, 0.001–100 μM) and slow-releasing H_2_S compounds (GYY4137, 0.001–10 μM) and the organosulfur H_2_S-releasing compounds, DADS (0.001–300 μM), SAC (0.1–1000 nM), and DATTS (0.001 pM − 1 μM), caused a concentration-dependent attenuation of LPS-induced increases in the levels of TNF-α, IL-6, and PGE_2_. DATTS (0.001 pM – 10 nM) reversed the LPS (25 ng/ml)-induced decrease in IL-10 production in the explants. We conclude that both fast- and slow-releasing H_2_S compounds, as well as polysulfides, were effective in reducing LPS-induced acute inflammation in porcine anterior uveal explants, *ex vivo*. Inhibition of endogenous H_2_S biosynthesis exacerbates LPS-induced production of an inflammatory mediator, a response that was reversed by an H_2_S-releasing compound.

## Background

Inflammation is a complex component of the immune system's response to harmful agents, resulting in the release of proinflammatory mediators such as tumor necrosis factor-alpha (TNF-α), prostaglandin E_2_ (PGE_2_), interleukin-6 (IL-6), and anti-inflammatory mediators such as interleukin-10 (IL-10) [1, 2]. Inflammation is a common feature of many diseases, such as sepsis, cardiovascular diseases (atherosclerosis), diabetes, arthritis, and uveitis [3–7]. Uveitis is an inflammatory condition involving the anterior uvea, as well as the adjacent structures in the eye [8, 9]. This disease accounts for 5–10% of visual impairment and is a significant cause of blindness around the world [3, 10]. While the etiology of uveitis is yet to be fully understood, infections, autoimmune disorders, and exposure to toxins have been reported to cause this disease [11, 12]. Although current strategies for the therapy of uveitis include the use of drugs such as corticosteroids, antimetabolites, alkylating agents, and biological agents, some side effects have been reported [13]. Consequently, the identification of alternate therapeutic targets for treating uveitis is warranted.

Hydrogen sulfide (H_2_S) is a gaseous molecule with extensive physiological and pathological actions (e.g., neuromodulation, cardio-protection, cell proliferation, apoptosis, etc.) in several tissues and organs [14]. The four major enzymes that are involved in the biosynthesis of H_2_S in the eye include cystathionine β-synthase (CBS), cystathionine γ-lyase (CSE), and 3-mercaptopyruvate sulfur transferase (3-MST) with cysteine aminotransferase (CAT) [15]. These enzymes are also present in ocular tissues such as the cornea, iris, ciliary body, and retina [15–17]. Evidence from our laboratory also confirmed the endogenous production of H_2_S in ocular tissues [18] and the pharmacological actions of H_2_S-releasing compounds (such as decreases in intraocular pressure and relaxation of the vasculature) in eyes from several species [18–31]. H_2_S has been reported to play a crucial role in inflammation in several mammalian tissues and organ systems [32, 33]. At low concentrations of H_2_S, this gas has been shown to possess anti-inflammatory actions [34–37]. However, there is some evidence that high concentrations of this gas can have pro-inflammatory actions in some systems [32, 38]. H_2_S-releasing compounds employed in biological studies are classified based on the rate of release of H_2_S gas. The most common class is the fast H_2_S-releasing compounds, which provide direct and instantaneous release of H_2_S and include the sulfide salts such as sodium hydrosulfide (NaHS) and sodium sulfide (Na_2_S) [39]. Use of synthetic compounds that release H_2_S slowly, including the Lawesson’s (phosphorodithioate) derivative such as GYY4137 have been widely used to evaluate the therapeutic potential of exogenous H_2_S delivery to tissues and/or organs [39]. Garlic (*Allium sativum*) is widely used due to its numerous health benefits, which can be attributed to its rich source of organosulfur compounds [40]. These naturally occurring compounds extracted from garlic include allicin, S-allyl cysteine, diallyl mono- and polysulfides, as well as methylated forms of allyl mono- and polysulfides are known to release H_2_S [41, 42]. Since ocular tissues can produce H_2_S [18, 43, 44], and there is evidence that this gas is involved in the inflammatory process [32, 38], the aim of the present study was: (1) to investigate the pharmacological actions of H_2_S (using exogenously administered as gas-releasing compounds) on LPS-induced inflammatory effects in a porcine *ex vivo* model of acute uveitis, and (2) to examine the role of endogenous H_2_S in the LPS-induced release of the inflammatory mediator, prostaglandin E_2_ in the absence and presence of the garlic-derived, H_2_S-releasing compound, S-allyl-cysteine (SAC) in this model. Some of the preliminary data presented in this paper have been published as Abstracts [45, 46].

## Methods

### Chemicals and Reagents

Lipopolysaccharide (LPS, Escherichia Coli Serotype O55:B5), Hanks’ balanced salt solution (HBSS), sodium hydrosulfide (NaHS), indomethacin, and enzyme-linked immunoassay kits for TNF-α and IL-6 were purchased from Sigma Chemical (St. Louis, MO). GYY4137, flurbiprofen, aminoaxyxacetic acid (AOAA), and enzyme-linked immunoassay kits for PGE_2_, as well as stock solutions of diallyl disulfide (DADS), S-allyl cysteine (SAC), and diallyl tetrasulfide (DATTS), were purchased from Cayman Chemical (Ann Harbor, MI). Roswell Parkland Institute Medium 1640 (RPMI 1640), Penicillin-Streptomycin, and enzyme-linked immunoassay kits for IL-10 were purchased from ATCC, Corning, and Thermofisher, respectively. All test agents were freshly prepared immediately before use in the series of experiments. Stock solutions of NaHS and GYY4137 were prepared in deionized water, while stock solutions of indomethacin and flurbiprofen were prepared in 100% DMSO.

### Tissue Preparation and Tissue Culture

Porcine eyes were obtained within 24 hours of euthanasia from First Vision Tech (Dallas, TX) and transported to the laboratory on ice. Eyeballs were dissected, and the irises with the ciliary body attached were carefully isolated. The methodology utilized for the preparation of tissues for *ex vivo* studies was essentially similar to that described by Brito et al. [47] using human iris-ciliary bodies, with some modifications. Briefly, isolated iris-ciliary body (ICB) explants were cut into four equivalent quadrants, maintained in oxygenated Krebs-Henseleit solution, and incubated in separate wells of a 24-well culture plate containing 500 μl of RPMI 1640 supplemented with 1% Penicillin-Streptomycin. Quadrants of the porcine ICB were randomly assigned to the control and treatment groups. LPS was reconstituted in HBSS to make a stock solution of 100 μg/ml, aliquoted, and stored at −20°C. Final concentrations of LPS, H_2_S-releasing compound (SAC), and the inhibitor of H_2_S biosynthesis (AOAA) were prepared in RPMI 1640 medium. Explants were treated with varying concentrations of LPS (5–200 ng/mL). When used, explants were treated with the fast H_2_S-releasing compound, NaHS, for 30 minutes before the end of the incubation period. For slow H_2_S-releasing compounds, GYY4137, DADS, SAC, and DATTS, the explants were treated for up to four hours before the end of the incubation period. In some experiments, explants were pre-treated with an inhibitor of H_2_S biosynthesis, AOAA (1 μM), or inhibitors of cyclooxygenase (COX), indomethacin (10 μM) and flurbiprofen (30 μM), for two hours before treatment with LPS. These inhibitors remained in culture media until the end of incubation. All explants were incubated at 37°C for 20 hours, and the culture media were collected, centrifuged, and assayed for their content of mediators, TNF-α, IL-6, PGE_2_, and IL-10 by enzyme-linked immunosorbent assay (ELISA) using commercially available kits according to the manufacturer’s instructions.

### Statistical Analysis

Results were expressed as either cytokine concentration per milligram soluble protein or as the percentage reduction in cytokine level, with values reported as means ± standard errors of the mean (mean ± S.E.M). The percentage reduction/increase for each cytokine was calculated based on the mediator level in tissues treated with H_2_S-releasing compounds in the presence of LPS when compared with tissues stimulated with the same concentration of LPS. IC_50_ values were calculated as the concentration of H_2_S-releasing compound that caused 50% inhibition of LPS-stimulated production of inflammatory mediators. Statistical analysis was performed using one-way ANOVA with Dunnett's test or two-way ANOVA with Tukey's post hoc analysis. Differences with P values < 0.05 were denoted as statistically significant. All data were analyzed using GraphPad Prism, version 10.2.3 (GraphPad Software Inc.).

## Results

In 2004, Brito and colleagues reported that in an explant culture of the human iris-ciliary body, LPS can induce the release of IL-6 and TNFα, a response that was inhibited by anti-toll-like receptor-4 monoclonal antibody, suggesting that the sensitivity of the anterior uvea to bacterial endotoxin could be used as a standard animal model of acute endotoxin-induced uveitis [47]. In the present study, we modified the experimental conditions employed by Brito et al. [47] using porcine iris-ciliary body explants, and indeed, obtained similar results when the explants were exposed to LPS.

### Effect of Lipopolysaccharide on the Release of Pro-inflammatory/Anti-inflammatory Mediators in Porcine Iris-ciliary Body Explants

**Figure 1:**
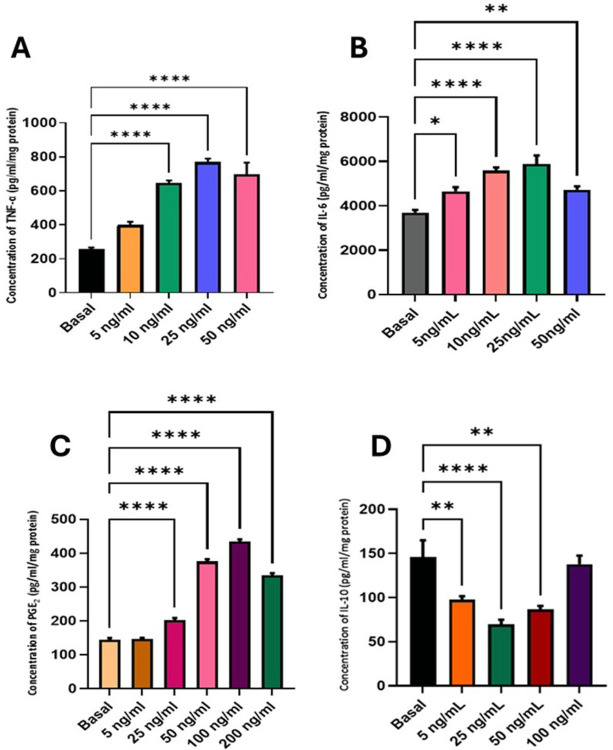
Effect of LPS (5 – 200 ng/ml) on the Release of Pro-inflammatory and Anti-inflammatory Mediators in Cultured Porcine Iris-ciliary Body Explants. Each value represents the mean ± SEM for (a) TNF-α, (b) IL-6, (c) PGE_2,_ and (d) IL-10. *p < 0.05, **p < 0.01, ****p < 0.001, significantly different from basal concentrations (n = 9 – 12).

In a series of experiments, we examined the effect of different concentrations of LPS on the release of inflammatory mediators, IL-6, TNF-α, and PGE_2_ in the porcine ICB explants. As shown in Fig. 1(A–C), incubation of porcine ICB explants with LPS (5 ng/ml – 200 ng/ml) caused a concentration-dependent significant (p < 0.05 to p < 0.001) increase in the release of IL-6, TNF-α, and PGE_2_ into the media of the porcine ICB explants after 20 hours of incubation. The basal concentrations of TNF-α, IL-6, and PGE_2_ were 258.6 ± 10.7 pg/ml/mg protein, 3695.6 ± 121.8 pg/ml/mg protein, and 144.3 ± 5.6 pg/ml/mg protein, respectively.

In another series of experiments, we measured the concentration of IL-10, an anti-inflammatory mediator, in the media containing porcine ICB explants. LPS (5 ng/ml – 100 ng/ml) produced a concentration-dependent decrease in IL-10 concentration that reached a maximum at 25 ng/ml. (Fig. 1D).

### Effects of Fast- and Slow-H_2_S-Releasing Compounds on Lipopolysaccharide-induced Release of Pro-inflammatory Mediators

**Figure 2:**
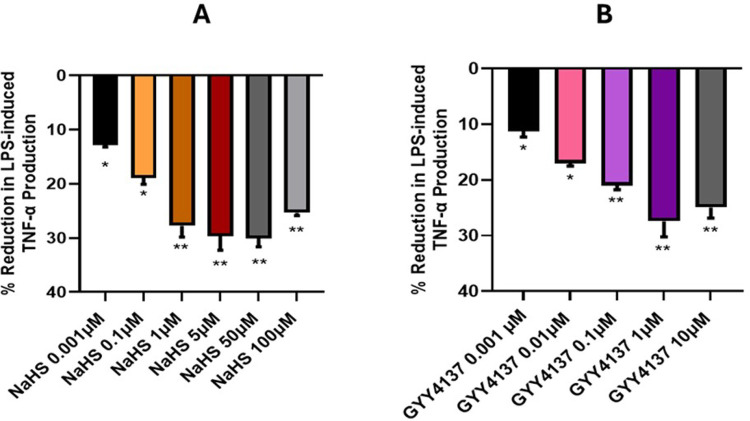
Effects of Fast- and Slow-Releasing H_2_S Compounds on LPS-induced TNF-α Production in Iris-ciliary Body Explants. Each value represents the mean ± SEM for (a) NaHS (0.001 μM - 100 μM), (b) GYY4137 (0.001 μM −10 μM). *p < 0.05; **p < 0.01, significantly different from stimulated TNF-α release by LPS 25 ng/ml (n = 12).

To assess the effects of fast- and slow-releasing H_2_S compounds on the production of pro-inflammatory mediators, porcine ICB explants were incubated with a fast (NaHS) and a slow (GYY4137) H_2_S-releasing compound. Incubation with NaHS (0.001 μM − 100 μM) caused a concentration-dependent reduction in TNF-α release induced by LPS (25 ng/ml), with the highest reduction achieved at a concentration of 5 μM (29.7% ± 1.5%) (Fig. 2A). Similarly, GYY4137 (0.001 μM − 10 μM) produced a concentration-related decrease in TNF-α release induced by LPS (25 ng/ml), with the highest reduction achieved at 1 μM (27.4% ± 2.8%) (Fig. 2B).

**Figure 3:**
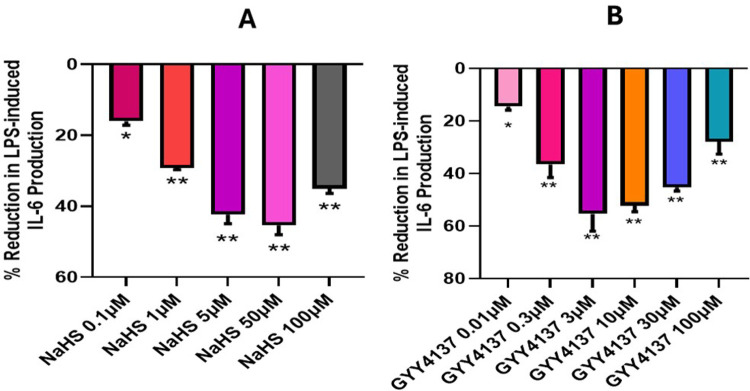
Effects of Fast- and Slow-Releasing H_2_S Compounds on LPS-induced IL-6 Production in Iris-ciliary Body Explants. Each value represents the mean ± SEM for (a) NaHS (0.1 μM - 100 μM) (b) GYY4137 (0.01 μM −100 μM). *p < 0.05; **p < 0.01, significantly different from stimulated IL-6 release by LPS 25 ng/ml (n = 9 – 12).

The fast-releasing H_2_S compound, NaHS (0.1 μM − 100 μM), also produced a concentration-dependent decrease in IL-6 production in the presence of LPS 25 ng/ml, with the most reduction observed at 50 μM (45.4% ± 2.7%) (Fig. 3A). Likewise, in the presence of LPS 25 ng/ml, GYY4137 (0.01 μM −100 μM) elicited a concentration-dependent decline in IL-6 release, reaching a maximum at 3 μM (55.4% ± 6.5%) (Fig. 3B).

**Figure 4:**
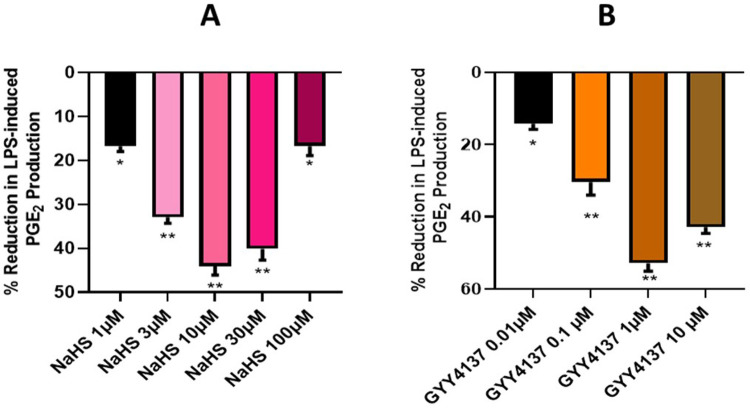
Effects of Fast- and Slow-Releasing H_2_S Compounds on LPS-induced PGE_2_ Production in Iris-ciliary Body Explants. Each value represents the mean ± SEM for (a) NaHS (1 μM - 100 μM) (b) GYY4137 (0.01 μM −10 μM). *p < 0.05; **p < 0.01, significantly different from stimulated PGE_2_ release by LPS 50 ng/ml (n = 9 – 12).

The fast-releasing H_2_S compound, NaHS (1 μM −100 μM) caused a concentration-related decrease in LPS (50 ng/ml)-induced PGE_2_ release in the porcine ICB explants, reaching a maximum at 10 μM (44.1% ± 2.0%) (Fig. 4A). Furthermore, GYY4137 (0.01 μM − 10 μM) elicited a concentration-dependent decrease in LPS (50 ng/ml)-induced PGE_2_ production, with the most reduction observed at a concentration of 1 μM (52.8% ± 2.2%) (Fig. 4B).

### Effects of Organosulfur HS-Releasing Compounds on Lipopolysaccharide-induced Release of PGE in Porcine Iris-ciliary Body Explants

**Figure 5:**
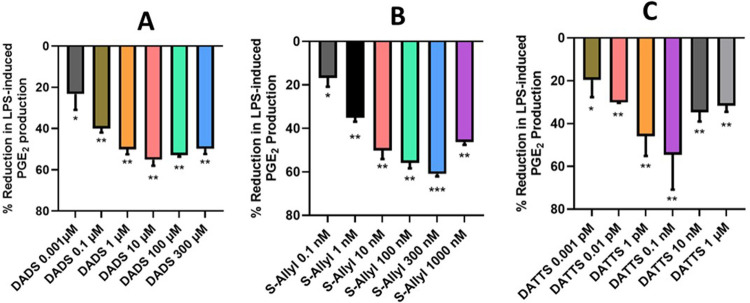
Effects of Organosulfur H_2_S-Releasing Compounds on LPS-induced PGE_2_ Production in Iris-ciliary Body Explants. Each value represents the mean ± SEM for (a) DADS (0.001 μM - 300 μM) (b) SAC (0.1 nM - 1000 nM) (c) DATTS (0.001 pM - 1 μM). *p < 0.05; **p < 0.01; ***p < 0.001, significantly different from stimulated PGE_2_ release by LPS 50 ng/ml (n = 9 – 12).

Garlic-derived H_2_S-releasing compounds have been shown to have anti-inflammatory properties in non-ocular tissues. To assess the effects of these H_2_S-releasing compounds on the release of PGE2, porcine ICB explants were incubated with diallyl disulfide (DADS), S-allyl cysteine (SAC), and diallyl tetrasulfide (DATTS) in the presence of LPS (50 ng/ml). DADS (0.001 μM − 300 μM) produced a concentration-related decrease in PGE_2_ release from the explants, with a maximum reduction reached at a concentration of 10 μM (55.1% ± 1.2%) (Fig. 5A). Similarly, SAC (0.1 nM − 1000 nM) and DATTS (0.001 pM − 1 μM) produced significant concentration-dependent decreases in PGE_2_ release, with the highest reduction achieved at 300 nM (60.9% ± 1.1%) and 0.1 nM (54.6% ± 7.3%) shown in Fig. 5B and Fig. 5C respectively.

When compared to fast- and slow-releasing H_2_S compounds, organosulfur compounds derived from garlic also inhibited the release of PGE_2_ stimulated by LPS. The IC_50_ values of the different H_2_S-releasing compounds in inhibiting the release of PGE_2_ stimulated by LPS are shown in Table 1.

Each IC_50_ value represents the mean ± SEM of the concentration of H_2_S-releasing compounds that caused 50% inhibition of LPS-stimulated PGE_2_ production (n = 9–12).

#### Effects of a Polysulfide on the Release of an Anti-inflammatory Mediator in Lipopolysaccharide-treated Porcine Iris-ciliary Body Explants

**Figure 6:**
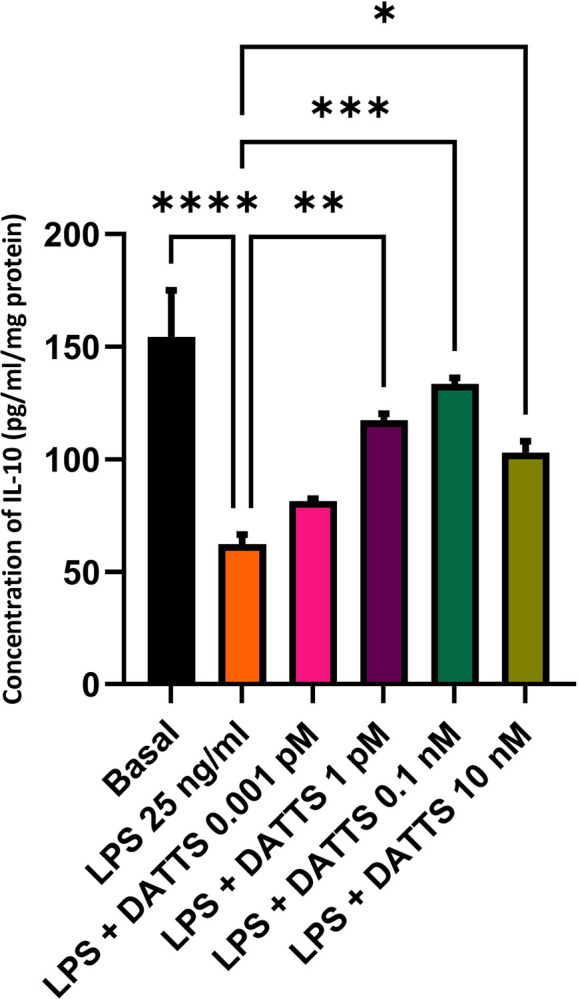
Effects of DATTS on LPS (25 ng/ml)-induced inhibition of IL-10 Production in Iris-ciliary Body Explants. Basal and in the presence of DATTS (0.001 pM – 10 nM). Each value represents the mean ± SEM. *p < 0.05; **p < 0.01; ***p < 0.001, significantly different from inhibited IL-10 release by LPS 25 ng/ml (n = 9).

We next assessed the effects of a polysulfide, DATTS, on the production of an anti-inflammatory mediator, IL-10, in the presence of LPS (50 ng/ml) in the porcine ICB explants. DATTS (0.001 pM – 10 nM) produced significant concentration-related reversal of LPS-induced decrease in IL-10 levels in the explants, reaching a maximum at a concentration of 0.1 nM (Fig. 6).

### Effect of inhibition of CBS/CSE on LPS-induced PGE production in Porcine Iris-ciliary Body Explants

**Figure 7:**
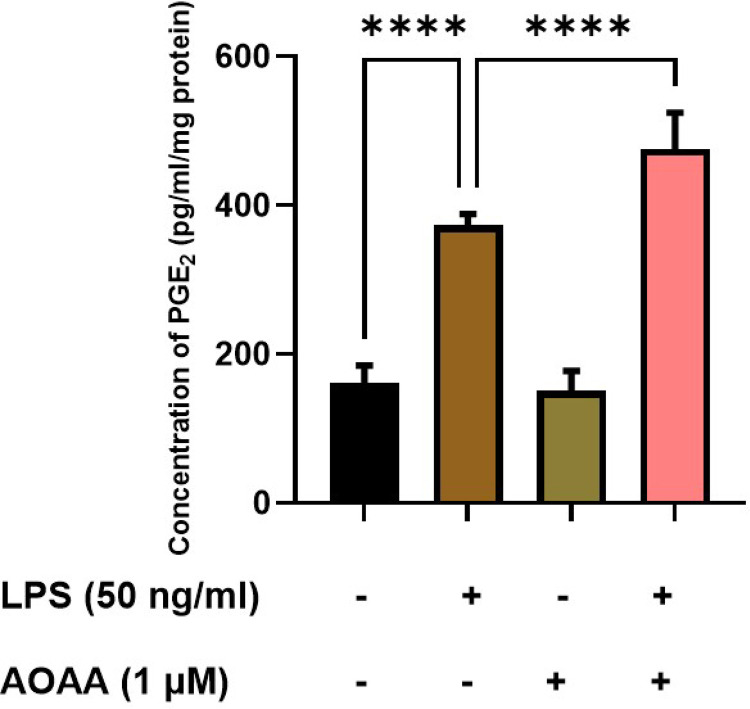
Effect of AOAA on LPS-Induced PGE_2_ Production in Cultured Porcine Iris-ciliary Body Explants. Each value represents the mean ± SEM. ****p < 0.0001, significantly different from stimulated PGE_2_ release by LPS 50 ng/ml (n = 8).

In a series of experiments, the effect of inhibition of CBS/CSE was examined on the LPS-induced inflammatory response. At a concentration of AOAA (1 μM) that had no significant action on basal PGE_2_ production from porcine ICB explants, LPS induced a significant (p < 0.001) increase in PGE_2_ release compared to LPS (50 ng/ml) alone (Fig. 7).

#### Effect of SAC on LPS-induced PGE_2_ Production in the presence of a CBS/CSE inhibitor in Porcine Iris-ciliary Body Explants

To assess the effect of SAC on LPS-induced PGE_2_ production in the presence of AOAA (1 μM), porcine ICB explants were incubated with a submaximal concentration of SAC (100 nM). SAC elicited a significant (p < 0.0001) reversal of the AOAA-induced elevation in the LPS-induced PGE_2_ release (Fig. 9).

### Effects of COX Inhibitors on Lipopolysaccharide-induced PGE_2_ Production in Porcine Iris-ciliary Body Explants

To assess the contribution of endogenous prostaglandins in the release of PGE_2_ induced by LPS (50 ng/ml) in the porcine ICB explants, tissues were incubated with the non-selective COX inhibitors, indomethacin (10 μM) and flurbiprofen (30 μM). In the absence of LPS, both indomethacin and flurbiprofen had no significant (p > 0.05) action on basal PGE_2_ concentrations (Table 3). In the presence of LPS-induced inflammation, both indomethacin and flurbiprofen significantly decreased PGE_2_ release by 56.3% ± 2.8% (n = 9) and 61.6% ± 6.4% (n = 9), respectively (Table 2).

Each value represents the mean ± SEM. *p < 0.05; **p < 0.01; ***p < 0.001, significantly different from stimulated PGE_2_ release by LPS 50 ng/ml (n = 9).

## Discussion

In 2004, Brito and colleagues developed a human ICB explant for studying acute anterior uveal inflammation using bacterial LPS as the insult and reported the release of pro-inflammatory mediators such as TNF-α and IL-6 into the culture media [47]. In the present study, we modified the protocol used by Brito et al. [47] for use with isolated porcine iris-ciliary bodies in culture. It is pertinent to note the use of cultured explants to study the inflammatory response triggered by LPS has been reported in human gestational tissues [48], lung explants [49], human abdominal aortic aneurysm explants [50], and adipose tissue [51]. Clearly, the approach used in the present study to investigate the pharmacological actions of H_2_S-releasing compounds in the porcine explants exposed to LPS supports previous work reported in the literature [48–51].

We found that treatment with different concentrations of LPS produced an increase in the release of TNF-α, IL-6, and PGE_2_ into the media, indicating that these mediators were associated with the acute inflammatory process. The use of LPS to induce inflammation in the anterior uvea has been reported in various experimental models of uveitis [48–54]. Indeed, LPS stimulated the production of pro-inflammatory cytokines such as TNF-α, IL-6, and PGE_2,_ which are commonly found in aqueous humor from patients with anterior uveitis and in experimental animals with EIU [55, 56]. It is pertinent to note that we were able to replicate the observation made by Brito et al. [47] on human ICB explants with the porcine tissue [47]. Taken together, data from the present study support the use of porcine tissues in studying inflammatory responses induced by LPS in the anterior uvea. The observation that the release of three distinct inflammatory mediators, TNF-α, IL-6, and PGE_2_, can be measured in the media containing the ICB explants, validates the use of these inflammatory markers for assessing anterior uveal inflammation.

Interestingly, in the present study, we observed that LPS caused a concentration-dependent decrease in basal levels of IL-10, an anti-inflammatory cytokine in the porcine ICB explants, suggesting that only processes that favor the onset and maintenance of inflammation prevail with the insult triggered by the bacterial LPS. Other investigators have also reported a decrease in the concentration of IL-10 during an inflammatory process [57, 58]. IL-10 is an essential anti-inflammatory multifunctional cytokine produced primarily from T cells and activated macrophages [59]. There is evidence that IL-10 can also inhibit the production of pro-inflammatory cytokines in tissues/organs [60, 61]. When compared to our earlier observations, it appears that as LPS stimulates the release of pro-inflammatory mediators (TNF-α, IL-6, and PGE_2_), it could be simultaneously suppressing the release of anti-inflammatory mediators such as IL-10. These findings are consistent with the studies reported by Vucevic et al., and other investigators which showed an inverse correlation between IL-6 and IL-10 levels in abdominal aortic aneurysm explant tissue cultures [50, 62]. In summary, data from the present study support the view that the bacterial LPS can initiate an inflammatory response in the porcine anterior uvea, *ex vivo*, a process that also inhibits the release of an anti-inflammatory cytokine such as IL-10.

There is abundant evidence of the anti-inflammatory role of H_2_S in several tissues and organs [63, 64]. Due to the difficulties associated with handling this gas for experimental purposes, H_2_S-releasing compounds serve as exogenous sources of this gas [65, 66]. In the present study, we examined the effects of fast- and slow-H_2_S-releasing compounds, as well as garlic-derived H_2_S-releasing compounds, on the production of pro- and anti-inflammatory mediators in the porcine ICB explants. Amongst the gas-releasing compounds, inorganic sulfide salts are the most widely used sources of exogenous H_2_S, with NaHS considered as the prototypical fast-releasing H_2_S compounds [39, 67]. The phosphorodithioate derivative, GYY4137 (morpholin-4-ium 4-methoxyphenyl-morpholino-phosphinodithioate) has been reported to demonstrate a slow-release H_2_S kinetics in biological media [68]. In a series of experiments, we observed that both NaHS and GYY4137 elicited concentration-dependent attenuation of LPS-induced increases in the levels of TNF-α, IL-6, and PGE_2_ in the culture media of porcine ICB explants, suggesting an anti-inflammatory role for both the fast- and slow-releasing H_2_S compounds. Our data also shows that at an equi-effective concentration of LPS (25 ng/ml), GYY4137 was more potent than NaHS in reducing the release of pro-inflammatory mediators in cultured porcine ICB explants. Our observation is consistent with results reported by Karaman et al. [67], where GYY4137 was shown to be up to 10 times more potent than NaHS in reducing LPS-stimulated TNF-α and IL-6 levels in an *in vitro* model of airway inflammation in mice.

Naturally occurring H_2_S-releasing compounds from garlic (such as S-allyl cysteine, DADS and DATTS) have been shown to release H_2_S in biological media at a relatively slow rate over time [69, 70, 71]. In a series of experiments, we investigated the effects of DADS, SAC, DATTS on LPS-induced PGE_2_ production in the porcine ICB explants. DADS, SAC, and DATTS elicited concentration-dependent decreases in the LPS-induced PGE_2_ production in the explants. We found that DATTS was more potent than SAC and DADS in decreasing PGE_2_ production at an equi-effective concentration of LPS (50 ng/ml). The observed discrepancy could be due to the ability of DATTS to release more H_2_S than DADS over time, since the reactivity of these organosulfur H_2_S-releasing compounds is directly related to the number of sulfur atoms present in the molecule [71, 72]. Attenuation of LPS-induced PGE_2_ production has also been reported for SAC and other diallyl compounds from garlic by other investigators [73–78]. The observed ability of SAC to prevent inflammation may be due to its action in releasing H_2_S since cysteine analogues such as S-propargyl-cysteine (SPRC) can increase levels of the gas in biological systems *via* mechanisms including direct H_2_S donation and upregulation of the expression of enzymes responsible for the biosynthesis of H_2_S [79, 80]. In the present study, the garlic-derived organosulfur compounds exhibited higher potencies in inhibiting the release of PGE_2_ stimulated by LPS when compared to fast- and slow-H_2_S-releasing compounds yielding the following rank order of potency: DATTS > > SAC > DADS > GYY4137 > NaHS.

We next examined the effect of DATTS on the production of IL-10, an anti-inflammatory cytokine, in the presence of LPS. We observed that DATTS significantly reversed the LPS-induced decrease in IL-10 levels in a concentration-dependent manner, indicating that DATTS can prevent the deleterious action of LPS on IL-10 production at the site of inflammation. Indeed, Flannigan et al. [80] demonstrated that a relationship exists between IL-10 and the pathway leading to H_2_S production since the administration of IL-10 to IL-10-deficient mice restored colonic H_2_S synthesis and reduced homocysteine levels. Be that as it may, the finding that DATTS released enough H_2_S to prevent the inhibitory action of LPS on the production of IL-10, supports the anti-inflammatory role of H_2_S in the porcine ICB explants.

SAC has been reported to act as a substrate for endogenous H_2_S biosynthesis in tissues and organs [79, 82]. We next investigated the role of an enzyme of the H_2_S trans-sulfuration pathway, CBS/CSE in the pharmacological effects produced by SAC on PGE_2_ production. There are several reports of the use of inhibitors of CBS/CSE such as AOAA and PAG (propargylglycine) to assess the pharmacological role of H_2_S in various cells, tissues, and organs [83, 84]. In the present study, we utilized AOAA to assess the role of CBS/CSE in the endogenous production of H_2_S during LPS-induced inflammation. We found that in the presence of AOAA, there was an enhancement of PGE_2_ release in LPS-treated porcine ICB explants, suggesting that inhibition of gas production further exacerbated the inflammation caused by LPS. Interestingly, treatment with SAC which releases H_2_S was able to reverse this response. These observations imply that endogenously produced H_2_S via a CBS/CSE-mediated pathway serves an anti-inflammatory role during the inflammation process. The replacement of depleted endogenous H_2_S content elicited by SAC was able to attenuate the inflammatory process and restore the anti-inflammatory action of the gas. A similar observation was made by Zhao et al. and other investigators who found that the inhibition of CBS activity by AOAA suppressed the protective effects of S-adenosyl-l-methionine (SAM) and H_2_S-releasing compounds on the secretion of pro-inflammatory cytokines due to astrocyte activation induced by LPS [85–88].

In another series of experiments, we sought to determine the source of PGE_2_ in the LPS-induced inflammatory response in porcine ICB explants. We investigated the effect of two COX inhibitors (indomethacin and flurbiprofen) on PGE_2_ levels in the LPS-induced inflammation model. In the presence of an equi-effective concentration of LPS (50 ng/ml), both indomethacin and flurbiprofen caused significant (p < 0.01) decreases in LPS-induced PGE_2_ release from the porcine ICB explants. The observed ability of COX inhibitors to attenuate inflammation-induced release of a mediator such as PGE_2_ confirms that active biosynthesis of endogenous prostaglandins contributes to this complex immune response.

A major significance of data obtained from the present study is that it supports the view that H_2_S-releasing compounds can inhibit the release of some mediators of inflammation. Data from the present study showing that SAC reversed the increased inflammation caused by the inhibition of an enzyme of the H_2_S trans-sulfuration pathway indicate that CBS/CSE activities could serve as a marker of inflammation in the pre-clinical and clinical settings [89]. A low expression or activity of these H_2_S trans-sulfuration enzymes demonstrated by low concentrations of the gas could indicate the presence of an inflammatory process in the anterior uvea. In support this assertion, evidence from our laboratory demonstrated that in porcine ICB explants, increasing concentrations of LPS caused a concentration-related decrease in the H_2_S levels indicating inflammation is associated with a decreased concentration of the gas [44]. Observations from the present study showing the ability of DATTS to prevent the decrease in IL-10 production induced by LPS, suggest that these gas-releasing compounds may have a potential therapeutic utility in enhancing the production of anti-inflammatory cytokines in uveitis.

## Conclusions

We conclude that the porcine ICB explants can serve as a model for studying inflammation induced by bacterial LPS. Furthermore, this model can be used to assess the effect of LPS on the release of both pro- and anti-inflammatory mediators in the anterior uvea. Both fast- and slow-releasing H_2_S compounds, as well as garlic-derived H_2_S-releasing compounds, were effective in reducing LPS-induced inflammation in porcine anterior uveal explants, *ex vivo*. Inhibition of the biosynthesis of H_2_S via the CBE/CSE and 3-MST pathways exacerbated the LPS-induced inflammation, a response that was reversed in the presence of a H_2_S-releasing compound. H_2_S-releasing compounds can also prevent the deleterious action of the bacterial LPS on the production of an anti-inflammatory cytokine in this model. The ability of LPS to increase the release of PGE_2_ in the explants was due, at least in part, to the endogenous biosynthesis of prostaglandins in the anterior uvea.

## Figures and Tables

**Figure 8:**
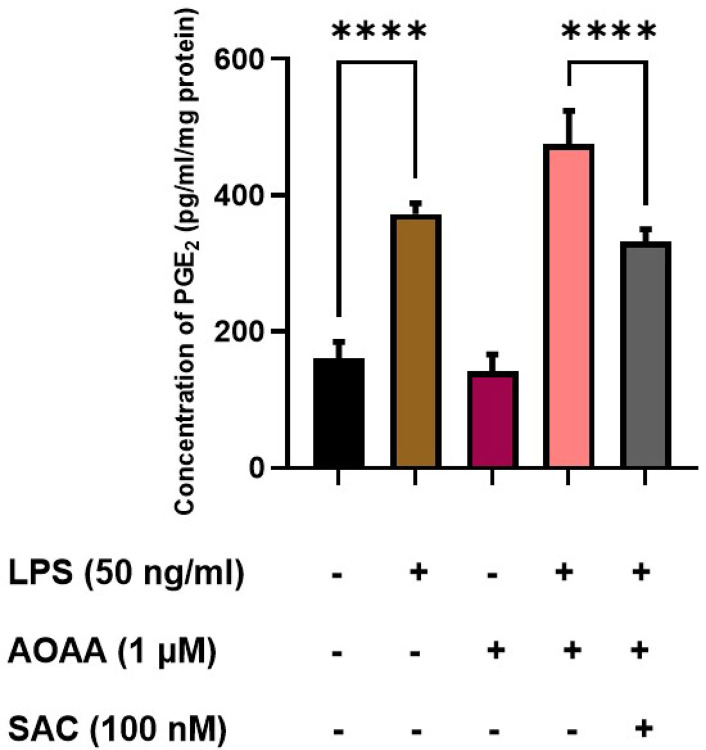
Effect of SAC on LPS-Induced PGE_2_ Production in Cultured Porcine Iris-ciliary Body Explants in the Presence of AOAA. Each value represents the mean ± SEM. ****p < 0.0001, significantly different from stimulated PGE_2_ release by LPS (50 ng/ml) in the presence of AOAA (n = 8).

**Table 1:** IC_50_ Values of H_2_S-Releasing Compounds in the Inhibition of LPS-induced PGE_2_ Production in Iris-ciliary Body Explants.

H_2_S-Releasing Compound	IC_50_ VALUES (nM)
NaHS	107.6 ± 79.5
GYY4137	74.4 ± 4.2
DADS	72.6 ± 5.8
SAC	1.19 ± 0.6
DATTS	0.015 ± 0.01

**Table 2:** Effects of COX Inhibitors on Lipopolysaccharide (LPS)-induced PGE_2_ Production in Iris-ciliary Body Explants.

TREATMENT	PGE_2_ CONCENTRATION (pg/ml/mg protein)
Basal	155.0 ± 14.6
LPS (50 ng/ml)	364.5 ± 3.8
Indomethacin (10 μM)	125.9 ± 11.5
LPS + Indomethacin (10 μM)	158.2 ± 2.8
Flurbiprofen (30 μM)	136.7 ± 6.5
LPS + Flurbiprofen (30 μM)	140.6 ± 6.4

Each value represents the mean ± SEM. *p < 0.05; **p < 0.01; ***p < 0.001, sig

## Data Availability

Datasets supporting the conclusions of this article are included within the article and its additional files.

## References

[1] Kolaczkowska E, Kubes P (2013) Neutrophil recruitment and function in health and inflammation. Nat Rev Immunol 13(3):159–175 23435331 10.1038/nri3399

[2] Sugimoto MA, Vago JP, Perretti M, Teixeira MM (2019) Mediators of the resolution of the inflammatory response. Trends Immunol 40(3):212–227 30772190 10.1016/j.it.2019.01.007

[3] Tsirouki T, Dastiridou A, Symeonidis C, Tounakaki O, Brazitikou I, Kalogeropoulos C, Androudi S (2018) A focus on the epidemiology of uveitis. Ocul Immunol Inflamm 26(1):2–16 27467180 10.1080/09273948.2016.1196713

[4] Nedeva C, Menassa J, Puthalakath H (2019) Sepsis: inflammation is a necessary evil. Front cell Dev biology 7:108 10.3389/fcell.2019.00108 PMC6596337 31281814

[5] Schett G (2019) November). Resolution of inflammation in arthritis. Seminars in immunopathology, vol 41. Springer Berlin Heidelberg, Berlin/Heidelberg, pp 675–679. 6 10.1007/s00281-019-00768-x 31720751

[6] Henein MY, Vancheri S, Longo G, Vancheri F (2022) The role of inflammation in cardiovascular disease. Int J Mol Sci 23(21):12906 36361701 10.3390/ijms232112906 PMC9658900

[7] Rohm TV, Meier DT, Olefsky JM, Donath MY (2022) Inflammation in obesity, diabetes, and related disorders. Immunity 55(1):31–55 35021057 10.1016/j.immuni.2021.12.013 PMC8773457

[8] Krishna U, Ajanaku D, Denniston AK, Gkika T (2017) Uveitis: a sight-threatening disease which can impact all systems. Postgrad Med J 93(1106):766–773 28942431 10.1136/postgradmedj-2017-134891

[9] Gilger BC, Degroote R, Deeg C (2022) Diseases of the uvea, uveitis, and recurrent uveitis. Equine Ophthalmol, 441–498

[10] Cunningham ET, Zierhut M (2021) Vision loss in uveitis. Ocul Immunol Inflamm 29(6):1037–1039 35040720 10.1080/09273948.2021.2017152

[11] Miserocchi E, Fogliato G, Modorati G, Bandello F (2013) Review on the worldwide epidemiology of uveitis. Eur J Ophthalmol 23(5):705–717. 10.5301/ejo.5000278 23661536

[12] Joltikov KA, Lobo-Chan AM (2021) Epidemiology and risk factors in non-infectious uveitis: a systematic review. Front Med 8:695904 10.3389/fmed.2021.695904 PMC8461013 34568364

[13] Babu K, Mahendradas P (2013) Medical management of uveitis - current trends. Indian J Ophthalmol 61(6):277–283. 10.4103/0301-4738.114099 23803479 PMC3744780

[14] Cirino G, Szabo C, Papapetropoulos A (2023) Physiological roles of hydrogen sulfide in mammalian cells, tissues, and organs. Physiol Rev 103(1):31–276 35435014 10.1152/physrev.00028.2021

[15] Gersztenkorn D, Coletta C, Zhu S, Ha Y, Liu H, Tie H, Motamedi M (2016) Hydrogen sulfide contributes to retinal neovascularization in ischemia-induced retinopathy. Investig Ophthalmol Vis Sci 57(7):3002–3009 27273718 10.1167/iovs.15-18555 PMC4904802

[16] Persa C, Osmotherly K, Chen C-W, Moon K, S., Lou MF (2006) The distribution of cystathionine betasynthase (CBS) in the eye: implication of the presence of a trans-sulfuration pathway for oxidative stress defense. Exp Eye Res 83(4):817–823. 10.1016/j.exer.2006.04.001 16769053

[17] Badiei A, Sudharsan R, Santana E, Dunaief JL, Aguirre GD (2019) Comparative localization of cystathionine beta synthases and cystathionine gamma lyase in canine, non-human primate and human retina. Exp Eye Res 181:72–84 30653965 10.1016/j.exer.2019.01.007 PMC6443508

[18] Kulkarni M, Njie-Mbye YF, Okpobiri I, Zhao M, Opere CA, Ohia SE (2011) Endogenous production of hydrogen sulfide in isolated bovine eye. Neurochem Res 36(8):1540–1545. 10.1007/s11064-011-0482-6 21533862

[19] Kulkarni KH, Monjok EM, Zeyssig R, Kouamou G, Bongmba ON, Opere CA, Njie YF, Ohia SE (2009) Effect of hydrogen sulfide on sympathetic neurotransmission and catecholamine levels in isolated porcine iris-ciliary body. Neurochem Res 34(3):400–406. 10.1007/s11064-008-97937 18629636

[20] Kulkarni-Chitnis M, Njie-Mbye YF, Mitchell L, Robinson J, Whiteman M, Wood ME, Opere CA, Ohia SE (2015) Inhibitory action of novel hydrogen sulfide donors on bovine isolated posterior ciliary arteries. Exp Eye Res 134:73–79. 10.1016/j.exer.2015.04.001 25845295 PMC4426029

[21] Monjok EM, Kulkarni KH, Kouamou G, McKoy M, Opere CA, Bongmba ON, Njie YF, Ohia SE (2008) Inhibitory action of hydrogen sulfide on muscarinic receptor-induced contraction of isolated porcine irides. Exp Eye Res 87(6):612–616 18940190 10.1016/j.exer.2008.09.011

[22] Njie-Mbye YF, Bongmba OY, Onyema CC, Chitnis A, Kulkarni M, Opere CA, LeDay AM, Ohia SE (2010) Effect of hydrogen sulfide on cyclic AMP production in isolated bovine and porcine neural retinae. Neurochem Res 35(3):487–494. 10.1007/s11064-009-0085-7 19898983

[23] Njie-Mbye YF, Kulkarni M, Opere CA, Ohia SE (2012) Mechanism of action of hydrogen sulfide on cyclic AMP formation in rat retinal pigment epithelial cells. Exp Eye Res 98:16–22. 10.1016/j.exer.2012.03.001 22445555

[24] Ohia SE, Opere CA, Monjok EM, Kouamou G, Leday AM, Njie-Mbye YF (2010) Role of hydrogen sulfide production in inhibitory action of L-cysteine on isolated porcine irides. Curr Eye Res 35(5):402–407. 10.3109/02713680903576716 20450253

[25] Opere CA, Monjok EM, Kulkarni KH, Njie YF, Ohia SE (2009) Regulation of [3H] D-aspartate release from mammalian isolated retinae by hydrogen sulfide. Neurochem Res 34(11):1962–1968. 10.1007/s11064-009-9984-x 19760175

[26] Robinson J, Okoro E, Ezuedu C, Bush L, Opere CA, Ohia SE, Njie-Mbye YF (2017) Effects of Hydrogen Sulfide-Releasing Compounds on Aqueous Humor Outflow Facility in Porcine Ocular Anterior Segments, Ex Vivo. J Ocul Pharmacol Ther 33(2):91–97. 10.1089/jop.2016.0037 28099049 PMC5333562

[27] Salvi A, Bankhele P, Jamil JM, Kulkarni-Chitnis M, Njie-Mbye YF, Ohia SE, Opere CA (2016a) Pharmacological Actions of Hydrogen Sulfide Donors on Sympathetic Neurotransmission in the Bovine Anterior Uvea. Vitro Neurochemical Res 41(5):1020–1028. 10.1007/s11064-015-1784-x 26700431

[28] Salvi A, Bankhele P, Jamil J, Chitnis MK, Njie-Mbye YF, Ohia SE, Opere CA (2016b) Effect of Hydrogen Sulfide Donors on Intraocular Pressure in Rabbits. J Ocul Pharmacol Ther 32(6):371–375. 10.1089/jop.2015.0144 27092593 PMC4960495

[29] Heruye SH, Mbye YF, Ohia SE, Opere CA (2022) Protective Action of Hydrogen Sulfide-Releasing Compounds against Oxidative Stress-Induced Cataract Formation in Cultured Bovine Lenses. Curr Eye Res 47(2):239–245. 10.1080/02713683.2021.1975764 34473602

[30] Bankhele P, Salvi A, Jamil J, Njie-Mbye F, Ohia S, Opere CA (2018) Neurochem Res 43(3):692–701. 10.1007/s11064-018-2471-5. Comparative Effects of Hydrogen Sulfide-Releasing Compounds on [3H]D-Aspartate Release from Bovine Isolated Retinae 29353375

[31] Chitnis MK, Njie-Mbye YF, Opere CA, Wood ME, Whiteman M, Ohia SE (2013) Pharmacological actions of the slow release hydrogen sulfide donor GYY4137 on phenylephrine-induced tone in isolated bovine ciliary artery. Exp Eye Res 116:350–354. 10.1016/j.exer.2013.10.004 24145109

[32] Whiteman M, Winyard PG (2011) Hydrogen sulfide and inflammation: the good, the bad, the ugly and the promising. Expert Rev Clin Pharmacol 4(1):13–32. 10.1586/ecp.10.134 22115346

[33] Manandhar S, Sinha P, Ejiwale G, Bhatia M (2021) Hydrogen Sulfide and its Interaction with Other Players in Inflammation. In: Zhu YC (ed) Advances in Hydrogen Sulfide Biology. Advances in Experimental Medicine and Biology, vol 1315. Springer, Singapore. 10.1007/978-981-16-0991-6_6. 34302691

[34] Whiteman M, Li L, Rose P, Tan CH, Parkinson DB, Moore PK (2010) The effect of hydrogen sulfide donors on lipopolysaccharide-induced formation of inflammatory mediators in macrophages. Antioxid Redox Signal 12(10):1147–1154 19769459 10.1089/ars.2009.2899 PMC2875982

[35] Li M, Li J, Zhang T, Zhao Q, Cheng J, Liu B, Wang C (2017) Syntheses, toxicities and anti-inflammation of H2S-donors based on non-steroidal anti-inflammatory drugs. Eur J Med Chem 138:51–65 28646655 10.1016/j.ejmech.2017.06.012

[36] Chen Y, Jin S, Teng X, Hu Z, Zhang Z, Qiu X, Wu Y (2018) Hydrogen sulfide attenuates LPS-induced acute kidney injury by inhibiting inflammation and oxidative stress. Oxidative Med Cell Longev 2018(1):6717212 10.1155/2018/6717212 PMC5831990 29636853

[37] Ciccone V, Piragine E, Gorica E, Citi V, Testai L, Pagnotta E, Martelli A (2022) Anti-inflammatory effect of the natural H2S-donor erucin in vascular endothelium. Int J Mol Sci 23(24):15593 36555238 10.3390/ijms232415593 PMC9778978

[38] Wallace JL, Ferraz JG, Muscara MN (2012) Hydrogen sulfide: an endogenous mediator of resolution of inflammation and injury. Antioxid Redox Signal 17(1):58–67. 10.1089/ars.2011.4351 22017247 PMC3342563

[39] Powell CR, Dillon KM, Matson JB (2018) A review of hydrogen sulfide (H2S) donors: Chemistry and potential therapeutic applications. Biochem Pharmacol 149:110–123 29175421 10.1016/j.bcp.2017.11.014 PMC5866188

[40] Yudhistira B, Punthi F, Lin JA, Sulaimana AS, Chang CK, Hsieh CW (2022) S-allyl cysteine in garlic (Allium sativum): Formation, biofunction, and resistance to food processing for value-added product development. Compr Rev Food Sci Food Saf 21(3):2665–2687 35355410 10.1111/1541-4337.12937

[41] Benavides GA, Squadrito GL, Mills RW, Patel HD, Isbell TS, Patel RP, Kraus DW (2007) Hydrogen sulfide mediates the vasoactivity of garlic. Proceedings of the National Academy of Sciences, 104(46), 17977–17982 10.1073/pnas.0705710104 PMC2084282 17951430

[42] Piragine E, Calderone V (2021) Pharmacological modulation of the hydrogen sulfide (H2S) system by dietary H2S-donors: A novel promising strategy in the prevention and treatment of type 2 diabetes mellitus. Phytother Res 35(4):1817–1846 33118671 10.1002/ptr.6923

[43] Mikami Y, Shibuya N, Kimura Y, Nagahara N, Yamada M, Kimura H (2011) Hydrogen sulfide protects the retina from light-induced degeneration by the modulation of Ca2 + influx. J Biol Chem 286(45):39379–39386. 10.1074/jbc.M111.298208 21937432 PMC3234762

[44] Okolie A, Nigro MR, Polk S, Stubbs K, Chelliah S, Ohia SE, Liang D, Mbye YF N (2024) Development and application of LC-MS/MS method for the quantification of hydrogen sulfide in the eye. Anal Biochem 687:115448 38158106 10.1016/j.ab.2023.115448 PMC11359680

[45] Ohia SE, Okolie A, Muili F, Opere CA, Njie-Mbye YF (2022) Effect of hydrogen sulfide-releasing compounds on lipopolysaccharide-induced inflammation in cultured porcine iris-ciliary body explants. Investig Ophthalmol Vis Sci 63(7):2123–F0139

[46] Okolie AC, Muili F, Ngele K, Ohia SE, Opere CA, Mbye YFN (2023) Hydrogen sulfide-releasing compounds decrease prostaglandin E2 production in cultured porcine iris-ciliary body explants exposed to lipopolysaccharide-induced inflammation. Investig Ophthalmol Vis Sci 64(8):5184–5184

[47] Brito BE, Zamora DO, Bonnah RA, Pan Y, Planck SR, Rosenbaum JT (2004) Toll-like receptor 4 and CD14 expression in human ciliary body and TLR-4 in human iris endothelial cells. Exp Eye Res 79(2):203–208. 10.1016/j.exer.2004.03.012 15325567

[48] Laham N, Brennecke P, Rice GE (1997) Interleukin-8 release from human gestational tissue explants: the effects of lipopolysaccharide and cytokines. Biol Reprod 57(3):616–620 9282999 10.1095/biolreprod57.3.616

[49] Zhang H, KIM YK, Govindarajan A, Baba A, Binnie M, Ranieri M, V., Slutsky AS (1999) Effect of adrenoreceptors on endotoxin-induced cytokines and lipid peroxidation in lung explants. Am J Respir Crit Care Med 160(5):1703–1710 10556144 10.1164/ajrccm.160.5.9903068

[50] Vucevic D, Maravic-Stojkovic V, Vasilijic S, Borovic-Labudovic M, Majstorovic I, Radak D, Colic M (2012) Inverse production of IL-6 and IL-10 by abdominal aortic aneurysm explant tissues in culture. Cardiovasc Pathol 21(6):482–489 22445549 10.1016/j.carpath.2012.02.006

[51] Starr ME, Saito M, Evers BM, Saito H (2015) Age-associated increase in cytokine production during systemic inflammation—II: the role of IL-1β in age-dependent IL-6 upregulation in adipose tissue. Journals Gerontol Ser A: Biomedical Sci Med Sci 70(12):1508–1515 10.1093/gerona/glu197 PMC4643612 25344820

[52] Yuan Z, Chen X, Yang W, Lou B, Ye N, Liu Y (2019) The anti-inflammatory effect of minocycline on endotoxin-induced uveitis and retinal inflammation in rats. Mol Vis 25:359 31354229 PMC6620367

[53] Park J, Kim JT, Lee SJ, Kim JC (2020) The anti-inflammatory effects of angiogenin in an endotoxin induced uveitis in rats. Int J Mol Sci 21(2):413 31936482 10.3390/ijms21020413 PMC7014170

[54] Xiao X, Liu Z, Su G, Liu H, Yin W, Guan Y, Yang P (2022) A novel uveitis model induced by lipopolysaccharide in zebrafish. Front Immunol 13:1042849 36532084 10.3389/fimmu.2022.1042849 PMC9751191

[55] Bonacini M, Soriano A, Cimino L, De Simone L, Bolletta E, Gozzi F, Croci S (2020) Cytokine profiling in aqueous humor samples from patients with non-infectious uveitis associated with systemic inflammatory diseases. Front Immunol 11:358 32210963 10.3389/fimmu.2020.00358 PMC7077343

[56] Errera MH, Pratas A, Fisson S, Manicom T, Boubaya M, Sedira N, Bloch-Queyrat C (2022) Cytokines, chemokines and growth factors profile in human aqueous humor in idiopathic uveitis. PLoS ONE, 17(1), e0254972 35061677 10.1371/journal.pone.0254972 PMC8782285

[57] Porro C, Cianciulli A, Panaro MA (2020) The regulatory role of IL-10 in neurodegenerative diseases. Biomolecules 10(7):1017 32659950 10.3390/biom10071017 PMC7407888

[58] Solleiro-Villavicencio H, Méndez-García LA, Ocampo-Aguilera NA, Baltazar-Pérez I, Arreola-Miranda JA, Aguayo-Guerrero JA, Escobedo G (2024) Decreased Hepatic and Serum Levels of IL-10 Concur with Increased Lobular Inflammation in Morbidly Obese Patients. Medicina 60(6):862 38929479 10.3390/medicina60060862 PMC11205754

[59] Liu C, Wang X, Cao X (2024) IL-10: A Key Regulator and potential therapeutic target in uveitis. Cell Immunol, 104885 39447525 10.1016/j.cellimm.2024.104885

[60] Ip WE, Hoshi N, Shouval DS, Snapper S, Medzhitov R (2017) Anti-inflammatory effect of IL-10 mediated by metabolic reprogramming of macrophages. Science 356(6337):513–519 28473584 10.1126/science.aal3535 PMC6260791

[61] Zhang Y, Yang Y, Song J, Yu W, Li Y, Liu D, Zheng Y (2024) Laoxianghuang polysaccharide promotes the anti-inflammatory cytokine interleukin-10 in colitis via gut microbial linoleic acid. Phytomedicine 135:156136 39454376 10.1016/j.phymed.2024.156136

[62] Sanchez-Correa B, Bergua JM, Campos C, Gayoso I, Arcos MJ, Bañas H, Tarazona R (2013) Cytokine profiles in acute myeloid leukemia patients at diagnosis: survival is inversely correlated with IL-6 and directly correlated with IL-10 levels. Cytokine 61(3):885–891 23357299 10.1016/j.cyto.2012.12.023

[63] Citi V, Martelli A, Brancaleone V, Brogi S, Gojon G, Montanaro R, Calderone V (2020) Anti-inflammatory and antiviral roles of hydrogen sulfide: Rationale for considering H2S donors in COVID-19 therapy. Br J Pharmacol 177(21):4931–4941 32783196 10.1111/bph.15230 PMC7436626

[64] Pushpakumar S, Kundu S, Weber G, Sen U (2021) Exogenous hydrogen sulfide and miR-21 antagonism attenuates macrophage-mediated inflammation in ischemia reperfusion injury of the aged kidney. Geroscience 43:1349–1367 33433751 10.1007/s11357-020-00299-6 PMC8190249

[65] Kashfi K, Olson KR (2013) Biology and therapeutic potential of hydrogen sulfide and hydrogen sulfide-releasing chimeras. Biochem Pharmacol 85(5):689–703 23103569 10.1016/j.bcp.2012.10.019 PMC3566320

[66] Levinn CM, Cerda MM, Pluth MD (2020) Activatable small-molecule hydrogen sulfide donors. Antioxid Redox Signal 32(2):96–109 31554416 10.1089/ars.2019.7841 PMC6918874

[67] Karaman Y, Kaya-Yasar Y, Bozkurt TE, Sahin-Erdemli I (2021) Hydrogen sulfide donors prevent lipopolysaccharide-induced airway hyperreactivity in an in vitro model of chronic inflammation in mice. Basic Clin Pharmacol Toxicol 128(5):652–660. 10.1111/bcpt.13551 33369105

[68] Li L, Whiteman M, Guan YY, Neo KL, Cheng Y, Lee SW, Moore PK (2008) Characterization of a novel, water-soluble hydrogen sulfide–releasing molecule (GYY4137) new insights into the biology of hydrogen sulfide. Circulation 117(18):2351–2360 18443240 10.1161/CIRCULATIONAHA.107.753467

[69] Predmore BL, Kondo K, Bhushan S, Zlatopolsky MA, King AL, Aragon JP, Lefer DJ (2012) The polysulfide diallyl trisulfide protects the ischemic myocardium by preservation of endogenous hydrogen sulfide and increasing nitric oxide bioavailability. Am J Physiol Heart Circ Physiol 302(11):H2410–H2418 22467307 10.1152/ajpheart.00044.2012 PMC3378306

[70] Cai YR, Hu CH (2017) Computational study of H2S release in reactions of diallyl polysulfides with thiols. J Phys Chem B 121(26):6359–6366 28609097 10.1021/acs.jpcb.7b03683

[71] Mhatre S, Anjali R, Sahai P, Auden J, Singh S, Mbye N, Y. F., Opere CA (2024) Glutathione Modulates Hydrogen Sulfide Release and the Ocular Hypotensive Action of Diallyl Polysulfide Compounds. Pharmaceuticals 17(10):1408 39459046 10.3390/ph17101408 PMC11510538

[72] Münchberg U, Anwar A, Mecklenburg S, Jacob C (2007) Polysulfides as biologically active ingredients of garlic. Org Biomol Chem 5(10):1505–1518 17571177 10.1039/b703832a

[73] Chang HP, Chen YH (2005) Differential effects of organosulfur compounds from garlic oil on nitric oxide and prostaglandin E2 in stimulated macrophages. Nutrition 21(4):530–536 15811776 10.1016/j.nut.2004.07.018

[74] Schafer G, Kaschula H, C (2014) The immunomodulation and anti-inflammatory effects of garlic organosulfur compounds in cancer chemoprevention. Anti-Cancer Agents Med Chem (Formerly Curr Med Chemistry-Anti-Cancer Agents) 14(2):233–240 10.2174/18715206113136660370 PMC3915757 24237225

[75] Jikah AN, Edo GI, Makia RS, Yousif E, Gaaz TS, Isoje EF, Umar H (2024) A review of the therapeutic potential of sulfur compounds in Allium sativum. Measurement: Food, 100195

[76] Torres-Reyes DU, Sánchez-Sánchez MA, de la Rocha C, Rojas-Mayorquín AE, López-Roa RI, Ortuño-Sahagún D, Carrera-Quintanar L (2024) Modulatory L-Alliin effect on acute inflammatory cytokines in diet-induced obesity mice. Metabolites 14(11):580 39590816 10.3390/metabo14110580 PMC11596104

[77] Khajevand-Khazaei MR, Azimi S, Sedighnejad L, Salari S, Ghorbanpour A, Baluchnejadmojarad T, Roghani M (2019) S-allyl cysteine protects against lipopolysaccharide-induced acute kidney injury in the C57BL/6 mouse strain: Involvement of oxidative stress and inflammation. Int Immunopharmacol 69:19–26 30665040 10.1016/j.intimp.2019.01.026

[78] Rousta AM, Mirahmadi SMS, Shahmohammadi A, Ramzi S, Baluchnejadmojarad T, Roghani M (2020) S-allyl cysteine, an active ingredient of garlic, attenuates acute liver dysfunction induced by lipopolysaccharide/d-galactosamine in mouse: Underlying mechanisms. J Biochem Mol Toxicol 34(9):e22518 32453893 10.1002/jbt.22518

[79] Wang Q, Wang XL, Liu HR, Rose P, Zhu YZ (2010) Protective effects of cysteine analogues on acute myocardial ischemia: novel modulators of endogenous H2S production. Antioxid Redox Signal 12(10):1155–1165 19842912 10.1089/ars.2009.2947

[80] Li M, Mao J, Zhu Y (2021) New therapeutic approaches using hydrogen sulfide donors in inflammation and immune response. Antioxid Redox Signal 35(5):341–356 33789440 10.1089/ars.2020.8249

[81] Flannigan KL, Agbor TA, Blackler RW, Kim JJ, Khan WI, Verdu EF, Wallace JL (2014) Impaired hydrogen sulfide synthesis and IL-10 signaling underlie hyperhomocysteinemia-associated exacerbation of colitis. Proceedings of the National Academy of Sciences, 111(37), 13559–13564 10.1073/pnas.1413390111 PMC4169975 25187563

[82] Bentke-Imiolek A, Szlęzak D, Zarzycka M, Wróbel M, Bronowicka-Adamska P (2024) S-Allyl-L-cysteine affects cell proliferation and expression of H2S-synthetizing enzymes in MCF-7 and MDAMB-231 adenocarcinoma cell lines. Biomolecules 14(2):188 38397425 10.3390/biom14020188 PMC10886539

[83] Papapetropoulos A, Whiteman M, Cirino G (2015) Pharmacological tools for hydrogen sulphide research: a brief, introductory guide for beginners. Br J Pharmacol 172(6):1633–1637 24909294 10.1111/bph.12806 PMC4369269

[84] Wang Y, Ni X, Chadha R, McCartney C, Lam Y, Brummett B, Xian M (2022) Methods for suppressing hydrogen sulfide in biological systems. Antioxid Redox Signal 36(4–6):294–308 34162216 10.1089/ars.2021.0088 PMC8865628

[85] Zhao Z, Guo W, Xu C, Wang Q, Mao C, Wan M (2023) Physiological functions and donor design of hydrogen sulfide and its application in central nervous system diseases. Chem Eng J 452:139089

[86] Xin D, Chu X, Bai X, Ma W, Yuan H, Qiu J, Wang Z (2018) l-Cysteine suppresses hypoxia-ischemia injury in neonatal mice by reducing glial activation, promoting autophagic flux and mediating synaptic modification via H2S formation. Brain Behav Immun 73:222–234 29751053 10.1016/j.bbi.2018.05.007

[87] Li TT, Xin DQ, Ke HF, Chu XL, Zhao YJ, Yue SW, Wang Z (2022) L-Cysteine attenuates osteopontin-mediated neuroinflammation following hypoxia-ischemia insult in neonatal mice by inducing S-sulfhydration of Stat3. Acta Pharmacol Sin 43(7):1658–1669 34737419 10.1038/s41401-021-00794-2 PMC9253102

[88] Lin T, Bai X, Gao Y, Zhang B, Shi J, Yuan B, Zhao X (2022) CTH/H2S Regulates LPS-Induced Inflammation through IL-8 Signaling in MAC-T Cells. Int J Mol Sci 23(19):11822 36233122 10.3390/ijms231911822 PMC9570289

[89] Bechelli C, Macabrey D, Deglise S, Allagnat F (2023) Clinical potential of hydrogen sulfide in peripheral arterial disease. Int J Mol Sci 24(12):9955 37373103 10.3390/ijms24129955 PMC10297923

